# Precision mapping child undernutrition for nearly 600,000 inhabited census villages in India

**DOI:** 10.1073/pnas.2025865118

**Published:** 2021-04-26

**Authors:** Rockli Kim, Avleen S. Bijral, Yun Xu, Xiuyuan Zhang, Jeffrey C. Blossom, Akshay Swaminathan, Gary King, Alok Kumar, Rakesh Sarwal, Juan M. Lavista Ferres, S. V. Subramanian

**Affiliations:** ^a^Division of Health Policy and Management, College of Health Science, Korea University, 02841 Seoul, South Korea;; ^b^Interdisciplinary Program in Precision Public Health, Department of Public Health Sciences, Graduate School of Korea University, 02841 Seoul, South Korea;; ^c^Harvard Center for Population and Development Studies, Cambridge, MA 02138;; ^d^Microsoft AI For Good Research Lab, Redmond, WA 98052;; ^e^SuperMap Software Co. Ltd, Beijing 100015, China;; ^f^Institute of Remote Sensing and Geographic Information System, Peking University, Beijing 100871, China;; ^g^Center for Geographic Analysis, Harvard University, Cambridge, MA 02138;; ^h^Flatiron Health, New York, NY 10012;; ^i^Institute for Quantitative Social Science, Harvard University, Cambridge, MA 02138;; ^j^Department of Medical Health and Family Welfare, Lucknow 226018, India;; ^k^National Institution for Transforming India Aayog, New Delhi 110001, India;; ^l^Department of Social and Behavioral Sciences, Harvard T.H. Chan School of Public Health, Boston, MA 02115;; ^m^National Institution for Transforming India Aayog, New Delhi 110001, India (Non-Resident)

**Keywords:** precision public policy, mapping, child undernutrition, local governance, India

## Abstract

There are emerging opportunities to assess health indicators at truly small areas with increasing availability of data geocoded to micro geographic units and advanced modeling techniques. The utility of such fine-grained data can be fully leveraged if linked to local governance units that are accountable for implementation of programs and interventions. We used data from the 2011 Indian Census for village-level demographic and amenities features and the 2016 Indian Demographic and Health Survey in a bias-corrected semisupervised regression framework to predict child anthropometric failures for all villages in India. Of the total geographic variation in predicted child anthropometric failure estimates, 54.2 to 72.3% were attributed to the village level followed by 20.6 to 39.5% to the state level. The mean predicted stunting was 37.9% (SD: 10.1%; IQR: 31.2 to 44.7%), and substantial variation was found across villages ranging from less than 5% for 691 villages to over 70% in 453 villages. Estimates at the village level can potentially shift the paradigm of policy discussion in India by enabling more informed prioritization and precise targeting. The proposed methodology can be adapted and applied to diverse population health indicators, and in other contexts, to reveal spatial heterogeneity at a finer geographic scale and identify local areas with the greatest needs and with direct implications for actions to take place.

## Significance

To the best of our knowledge, this is the first prediction of child anthropometric failure estimates for 597,121 villages—the smallest local governance unit—in India. While prior child nutrition policies and programs in India focused on districts for planning, implementation, and monitoring, we highlight that a majority of the geographic variation in child anthropometric failures occur at the micro geographic level of villages followed by the macro administrative level of states. Precision mapping of health data at the village level can enable more informed and effective local politics in India.

National trends in population health and development are now routinely available to guide policymaking even for most low- and middle-income countries (1). More recently, there has been an increasing recognition that national averages are inadequate given the substantial heterogeneity in patterns of disease and risk factors within any given country (1–3). As a consequence, there is a great interest for disaggregated data on population health and well-being to be provided and analyzed at subnational levels (1–3). Most studies that investigate subnational levels, however, are largely confined to macro geographies, such as states or districts in India (4, 5) or provinces in China (6), despite recent studies emphasizing more variation at finer geographic resolutions as small as villages or communities (7, 8). With increasing availability and accessibility of data geocoded to smaller geographic units and with varying degrees of precision along with the use of advanced modeling techniques, there are emerging opportunities to assess health and developmental indicators at truly small areas (9, 10). The future iterations of the Global Burden of Diseases, Injuries, and Risk Factors study are expected to feature maps of the different burden at a finer spatial resolution (5 × 5 km) (11). Geospatial analysis of estimates by 5 × 5 km grids has been presented for child mortality (12), child growth failure (13), childhood diarrheal morbidity and mortality (14), and women’s educational attainment (15) in Africa, and they revealed striking inequities at the local level.

While this interest toward a focus on finer geographic resolution is a welcoming step toward precision public health (2, 3, 9, 10, 16), small area estimates with no explicit link to political or administrative jurisdiction have limited practical implications in terms of guiding efficient and equitable interventions. To enable immediate attention and action to take place, the unit of analysis and inferential target in empirical studies need to align with the local governance unit, often within districts or cities, that are accountable for implementation of programs and interventions (3, 16, 17). Such fine-grained data are critical to identify and target areas with the greatest needs for prioritization, incorporate specific local needs and resource base for plan formulation, and appropriately evaluate the successes and failures of programs and policies at the local level (18).

With this conceptual motivation, and to aid the current movement toward decentralized planning in India to achieve global and national targets for population health and development, we present comprehensive estimates of child anthropometric failure for nearly 600,000 villages in rural India.

## Child Anthropometric Failures in India

India contributes to almost one-third of the global prevalence in stunting (5). Within India, child and maternal malnutrition remains the leading risk factor accounting for almost 15% of the total lost disability-adjusted life years (DALYs) (4). In addition to the Sustainable Development Goal (SDG) Target 2.2, which calls for an end to all forms of malnutrition by 2030 (19), the Government of India has declared the National Nutrition Mission (NNM or the Prime Minister’s Overarching Scheme for Holistic Nutrition Abhiyaan) with specific national targets of reducing child undernutrition by at least 2% per annum (20). Child anthropometric failure resulting from poor nutrition is associated with increased risk of morbidity and mortality, delayed motor and cognitive development, and lowered educational achievement and economic productivity in adulthood (21).

## Precision Policy Unit in the Indian Context

In India, almost all the states have populations larger than a typical country, with Uttar Pradesh being more populous than Brazil (22). In fact, even districts, on average, have about a 1.3 million rural population (23), making district-specific findings difficult to interpret in light of the substantial variation within districts (7, 8). Recent multilevel analyses on household poverty (7), catastrophic health spending (24), adult women’s body mass index (8), and child sex ratio (25) in India have all found a majority of geographic variation attributed to villages as opposed to the conventional macro units of districts or states. Moreover, the magnitude of between-village variation in these diverse outcomes were found to be heterogeneous across Indian states/union territories, with differential amounts being explained by covariate adjustments, indicating the need to explore specific mechanisms operating at the village level (7, 8, 24).

These findings can be interpreted in light of India’s Panchayati Raj system, which provides constitutional status to rural (village Panchayat) and urban (municipalities) local governments (18, 26). Since the constitutional framework for decentralized rural governance in India formalized in the 72nd Constitutional amendment in 1992, the Gram Panchayat serves as the unit of local government and is usually composed of one or more villages (27–29). The Gram Panchayat members are elected representatives who are responsible for overseeing local administrations, setting economic goals for their villages, demanding action from functionaries of various government departments, and serving as the channel for government assistance (28, 29). This form of local self-government fosters collective actions and cooperation with higher level government authorities at district or state levels (28, 29). The Panchayat system plays critical roles in preparation of plans for economic development and social justice and implementation of various developmental programs, including those concerning health and nutrition and disaster management (27, 30, 31). The importance of this grassroots level is expected to increase even more with the current political movement toward further development of mechanisms for village-level plans to be aggregated progressively at higher levels of government (18).

**Fig. 1.**
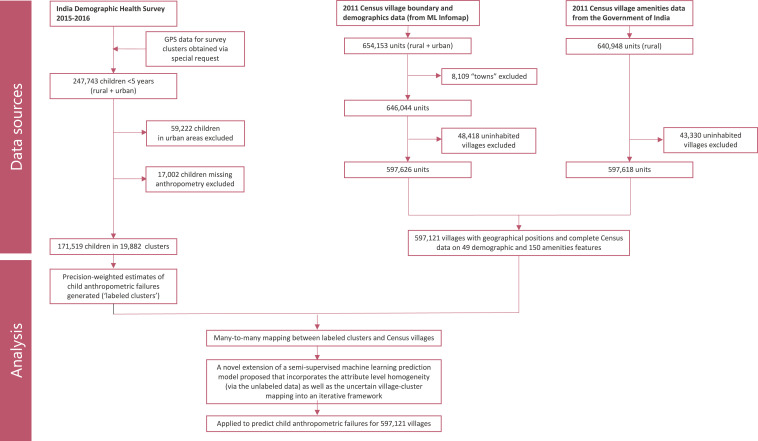
Flow diagram showing different data sources and analytics used to predict village estimates.

In this paper, we used several different data sources and novel methodologies to generate estimates of child anthropometric failures for all villages in India (Fig. 1). The 2011 Census for village-level demographic and amenities data were merged, resulting in 597,121 villages with geographical positions and complete Census attributes (“features”). Of note, the number of villages in India vary across different database, ranging anywhere between 600,000 and one million. Another data source we used was the 2016 Indian Demographic and Health Survey (DHS) with randomly displaced global positioning system (GPS) locations of survey clusters (equivalent to villages in Census). The DHS includes a nationally representative sample of children, and we generated precision-weighted estimates of child anthropometric failures (“labels”) for 19,882 clusters.

Since the GPS locations of these clusters were randomly displaced within a 5 km buffer, it was not possible to exactly identify the corresponding Census village for all 19,882 clusters. That is, there were several DHS clusters for which there were more than one possible corresponding Census village and several Census villages for which there were more than one possible corresponding DHS cluster. The resulting “many-to-many” relationship between the Census villages and survey clusters precluded direct application of a standard supervised regression method with a fixed label as a response variable and village features as covariates. Even if it were not the case, the number of survey clusters is likely insufficient to account for the heterogeneity and scale of villages in India. Moreover, nearly ∼45% of all villages had no association with a survey cluster. However, there is homogeneity at the attribute level, and we expect that villages with similar attributes to have comparable estimates of the quantities of interest. Thus, a method that exploits this assumption using the unlabeled village features and a relatively small amount of labeled data are relevant here.

Consequently, we propose an extension of a semisupervised machine learning prediction model that incorporates the attribute-level homogeneity (via the unlabeled data) as well as the uncertain village cluster mapping into an iterative framework. In general, our approach provides a way to extend small sample surveys (perhaps with some form of anonymization) to the entire population, making it relevant in other applications. We applied this methodology to predict child anthropometric failures for 597,121 inhabited villages in India. In the following sections, we provide an empirical assessment of the sources of geographic variation in child anthropometric failure and summarize the predicted estimates in terms of distribution across states and districts and by relevant geo-visualizations.

## Results

### Establishing the Significance of Villages as a Precision Policy Unit.

We assessed the relative importance of village level (compared to states and districts) in a multilevel model partitioning the total geographic variation in each child anthropometric failure outcome by multiple micro and macro levels. When random effects for village (level one), district (level two), and state (level three) were simultaneously considered, most of the variation in child anthropometric failure outcomes were consistently found to be at the village level (Table 1). For stunting, 68.9% of the total variation was attributed to villages, followed by 24.0% to states and around 7% at districts. More than half (54.2%) of the variation in underweight was attributed to villages and the remaining variation was attributed to states (39.5%) and districts (6.2%). Similarly, villages accounted for 72.3% of the variation in wasting.

**Table 1.** Partitioning total variation in predicted child anthropometric failures by village, district, and state levels

	Stunting %		Underweight %		Wasting %	
	Variance (SE)	Variance partitioning coefficient (%)	Variance (SE)	Variance partitioning coefficient (%)	Variance (SE)	Variance partitioning coefficient (%)
State	27.6 (7.0)	24.0%	56.5 (14.0)	39.5%	17.6 (4.5)	20.6%
District	8.2 (0.5)	7.1%	8.9 (0.5)	6.2%	6.1 (0.4)	7.1%
Village	79.3 (0.1)	68.9%	77.5 (0.1)	54.2%	61.9 (0.1)	72.3%
Total geographical variation	115.1	100%	142.9	100%	85.6	100%

Variance partitioning coefficient (%) for level *z* calculated as: $\frac{\sigma_{z}^{2}}{\sigma_{state}^{2}+\sigma_{District}^{2}+\sigma_{Village}^{2}}$ × 100.

### Precision Geo-Mapping of Child Anthropometric Failures.

**Fig. 2.**
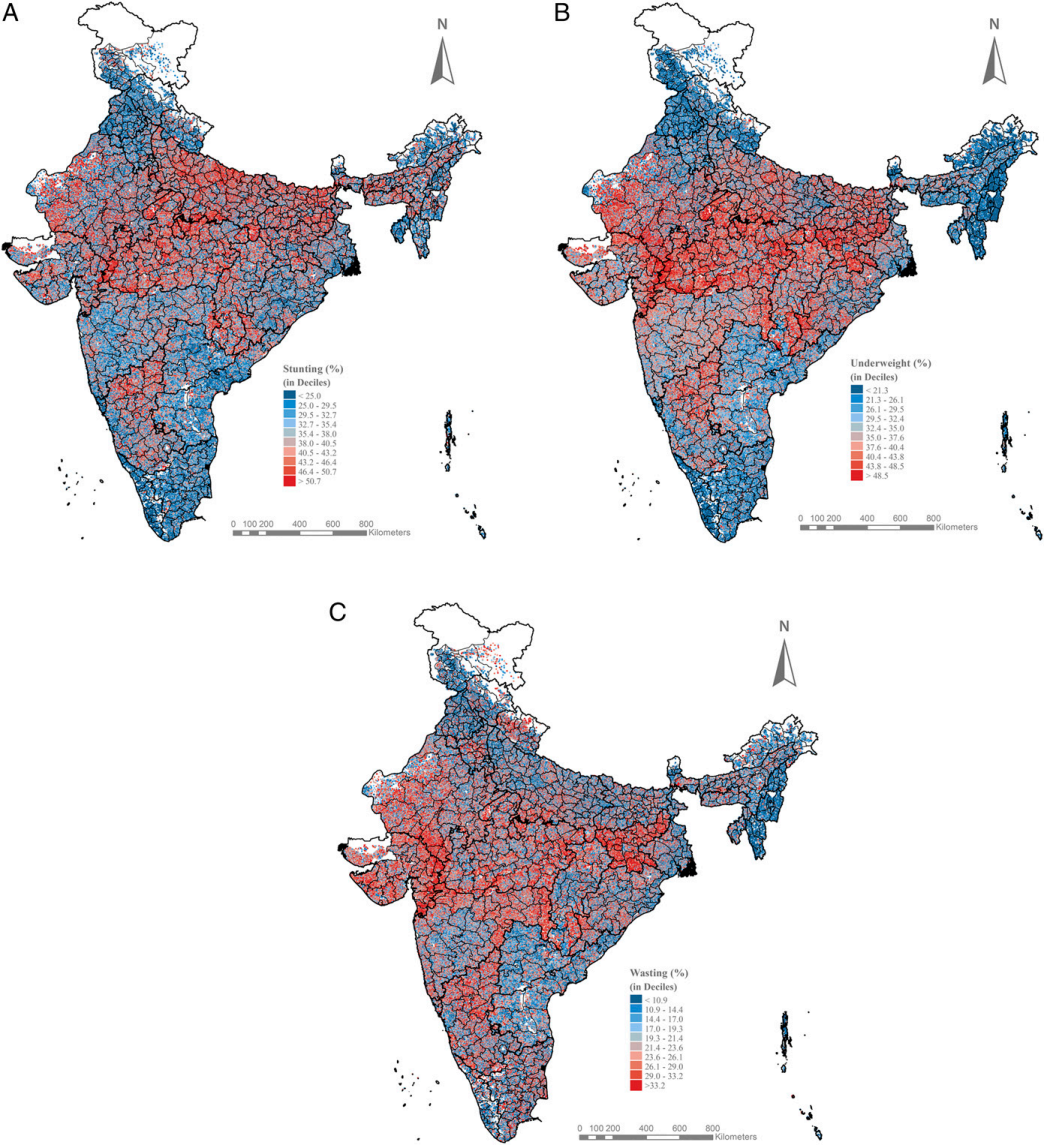
Maps showing village-level geography of predicted (*A*) stunting, (*B*) underweight, and (*C*) wasting across 597,121 villages in India.

The local geography of child anthropometric failures was assessed by mapping the predicted estimates for 597,121 villages across all of India (Fig. 2). The predicted estimates are mapped in deciles, with the lowest burden areas in dark blue to the highest burden areas in dark red. The predicted estimates are presented in Dataset S1, in which the villages are ranked from the highest to the lowest burden within district, within state, and at a national level. We also provide an interactive view of the village maps in a dashboard where users can view the predicted child anthropometric failures for a selected district: https://tiny.cc/IndiaVillage.

Visual inspection of the village maps indicated substantial variation across villages for child stunting, underweight, and wasting (Fig. 2). The mean predicted stunting was 37.9% (SD: 10.1%; interquartile range [IQR]: 31.2 to 44.7%), and it ranged from less than 5% for 691 villages to over 70% in 453 villages. Underweight was also highly prevalent in India, with the overall mean predicted estimate of 34.9% (SD = 10.7%; IQR: 27.9 to 42.0%) and high burden villages located in central and northern regions. Across all villages in rural India, 21.8% of the children were estimated to experience wasting (SD: 8.8%; IQR: 15.8 to 27.4%), a measure of acute undernourishment. At the same time, geographic clustering of villages with a high burden of child undernutrition was observed at the state level to some extent. For instance, areas with a high burden of stunting were concentrated especially in central and eastern regions of India.

### Village Variation by States and Districts.

#### Stunting.

**Fig. 3.**
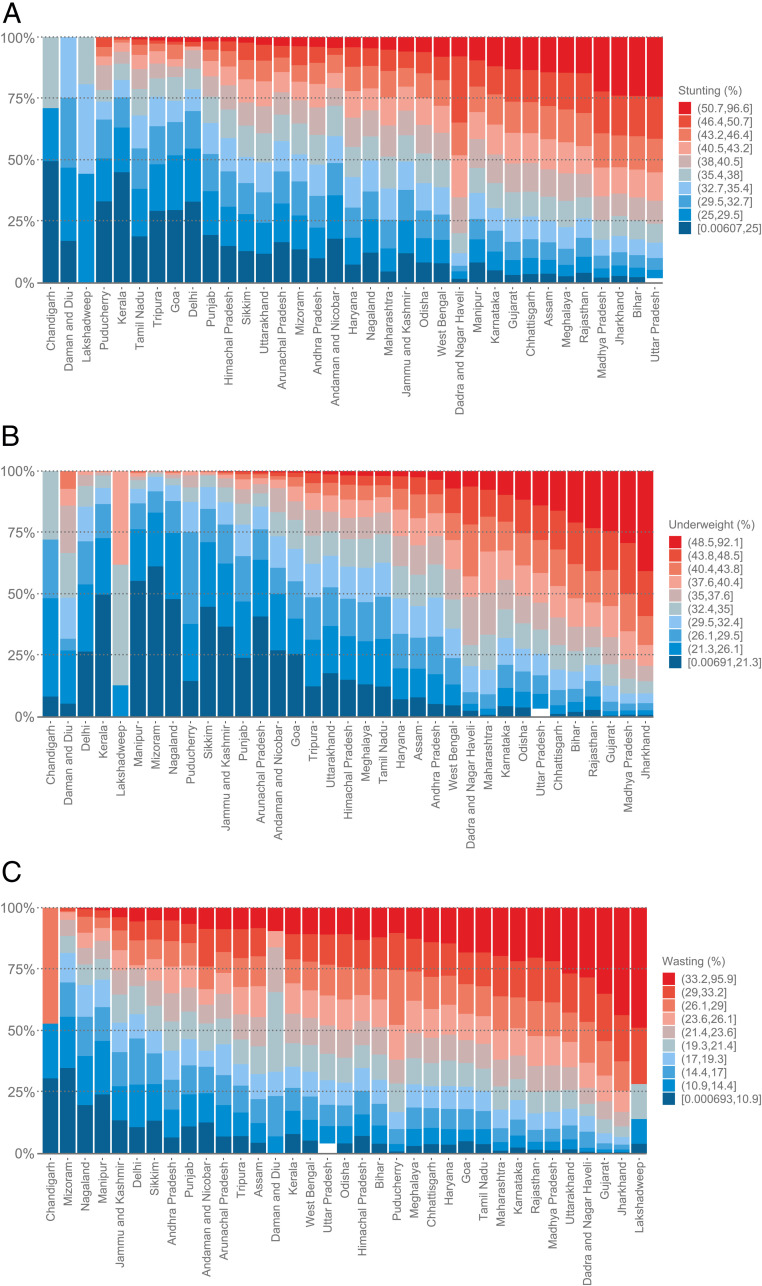
Stacked bars of villages in national deciles of (*A*) stunting, (*B*) underweight, and (*C*) wasting distributed across 36 states and union territories in India.

The variation in stunting was consistently large across and within all states, with means ranging from 22.1% in Kerala to 42.3% in Uttar Pradesh and SDs ranging from 4.0% in Lakshadweep to 10.1% in Jharkhand (Fig. 2*A*). Of the highest burden villages (i.e., top 10 percentile or ≥50.7% stunting), half of them were concentrated in three states of Uttar Pradesh (30.1%), Madhya Pradesh (14.5%), and Bihar (11.7%). In Uttar Pradesh, a state with 97,810 villages and one of the highest stunting prevalences (42.3%), almost a third of the villages had an estimate lower than the national mean (Fig. 3*A*). Villages with the lowest 10th percentile of child stunting (i.e., <25.0%) were concentrated in states of Odisha (11.5%), West Bengal (8.9%), Andhra Pradesh (7.2%), Himachal Pradesh (7.2%), and Tamil Nadu (7.1%). Except for smaller states of Chandigarh, Daman and Diu, and Lakshadweep, high- and low-burden villages coexisted within all states.

**Fig. 4.**
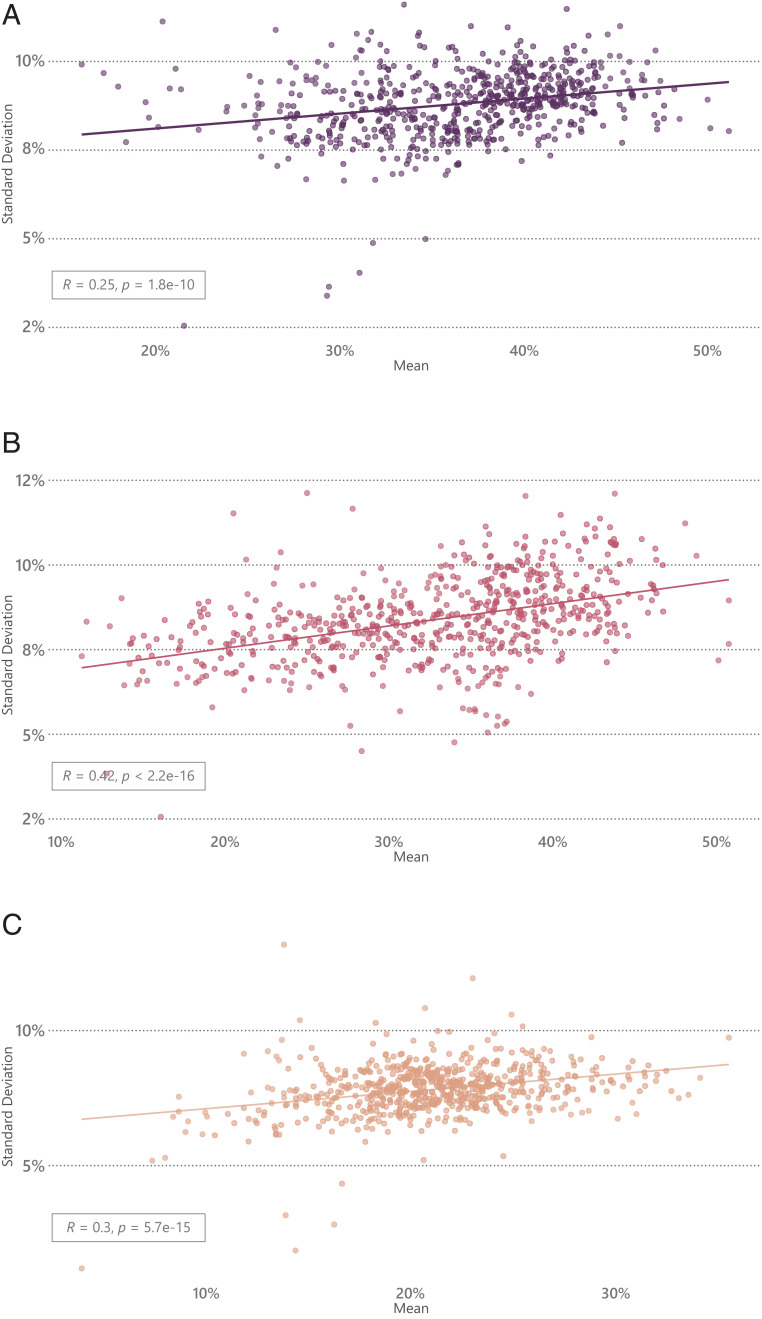
Correlation between district-wide mean and SD in (*A*) stunting, (*B*) underweight, and (*C*) wasting in India.

The variation in district-wide stunting was larger, with means ranging from 16.0% in Alappuzha (in Kerala) to 51.1% in Jhabua (in Madhya Pradesh) and intradistrict variation (SD) in stunting ranging from 3.4% in Daman district of Daman and Diu to 11.7% in Thoubal district in Manipur. District-wide mean and SD in predicted stunting had a weak correlation (*r* = 0.25) (Fig. 4*A*). In the district of Kolasib (in Mizoram), which had a stunting prevalence (33.5%) close to the national mean but the largest intradistrict variation (SD: 11.6%; IQR: 28.3 to 42.9%), the village-level prediction ranged from 11.4% in Hortoki to 55.9% in Bukvannei.

#### Underweight.

The state of Jharkhand (43.4%) had the highest prevalence of underweight, followed by Madhya Pradesh (41.8%) and Gujarat (40.4%). The intrastate variation in village estimates was the largest in Jharkhand (SD = 10.6%; IQR: 36.2 to 50.7%), Rajasthan (SD = 10.6%; IQR: 30.2 to 45.5%), and Bihar (SD = 10.0%; IQR: 31.8 to 45.0%) (Fig. 2*B*). Of the villages with the highest 10 percentile of predicted underweight (≥48.5%), 19.7% were located in Madhya Pradesh, 15.8% in Jharkhand, 15.4% in Uttar Pradesh, and 11.9% in Rajasthan. Even within Madhya Pradesh, a wide variation was observed for predicted underweight with 10% of the villages having low burden (i.e., underweight <30%) and another 13% of the villages having predicted underweight within the range of 30 to 35% (Fig. 3*B*).

A substantial variation was also found in district-wide underweight: means ranged from 11% in Chandel district of Manipur to over 50% in Dohad district of Gujarat and Jhabua district of Madhya Pradesh. Intradistrict variation in underweight ranged from SD = 3.8% in Rangareddy district of Andhra Pradesh to SD = 12.1% in Diu district of Daman and Diu. A moderate correlation was found for district-wide mean and SD in predicted underweight (*r* = 0.42), meaning that districts with a higher prevalence of underweight also tended to experience greater disparity (Fig. 4*B*). In the district of Palamu (in Jharkhand), which had one of the highest mean underweight (43.8%), around 25% of the villages had a prevalence lower than the national average while another 10% had a substantially high burden of underweight (>59%).

#### Wasting.

Across states, the mean predicted wasting ranged from 9.2% in Mizoram to 29.3% in Jharkhand, and the SD of wasting ranged from 5.2% in Daman and Diu to 11.9% in Lakshadweep (Fig. 2*C*). 16.2% of the highest burden villages (i.e., top 10 percentile or >33.2% wasting) were in the state of Jharkhand, followed by 12.2% in Madhya Pradesh and almost 10% each in Uttar Pradesh and Rajasthan. However, even within these states with high wasting, there were many villages with a relatively low burden (Fig. 3*C*). Within Jharkhand, for example, almost 20% of the villages had predicted wasting lower than the national median, or <21.4%.

There were 10 districts that had very low wasting (<10%) and 21 districts that had wasting >30%. The intradistrict village variation in wasting ranged from SD = 1.2% in Hyderabad district of Andhra Pradesh to SD = 11.9% in Lakshadweep district. There was a weak correlation between district-wide mean and SD in predicted wasting (*r* = 0.3) (Fig. 4*C*). In the district of Theni in Tamil Nadu, with a mean wasting of 24.9%, 8 villages (out of 80) had wasting <10%, 17 had 10% < wasting < 20%, 29 had 20% < wasting < 30%, 21 had 30% < wasting < 40%, and 5 had wasting >40%.

## Discussion

We utilized data from several sources and adopted a bias-corrected semisupervised regression to present an estimation of child anthropometric failures for nearly 600,000 villages in rural India. Our work highlights substantial variation across villages in child anthropometric failure that has been overlooked in prior literature. Specifically, we detected the following patterns, each with important implications for policies and interventions. First, we identified geographic patches of villages with a high burden of all three types of anthropometric failures. These areas represent the population with the greatest need and should be prioritized for interventions and monitored over time for progress. Second, these geographic patches of high burden villages were clustered at the state level to some extent but were not strictly contained within state boundaries, suggesting opportunities for interstate collaboration among contiguous local administrative units that share similar needs. Third, a mix of villages with high and low levels of burden exists in any given district. Only a weak to moderate correlation was found between district-wide mean and SD in child undernutrition. In the presence of substantial within-district variation in child undernutrition, aggregated estimates at the district level are less informative for policymaking. This level of heterogeneity supports that targeted nutrition and health programs tailored to the local needs will likely be more efficient than the traditional one-size-fits-all approaches.

Child anthropometric failures are caused by a complex etiology of proximate and distal risk factors, including inadequate dietary intake of key nutrients, exposure to infectious diseases, and socioeconomic factors that operate at multiple levels (21, 32, 33). Hence, improvement in child undernutrition at the population level necessitates a co-occurrence of nutrition-specific interventions to promote dietary diversity and appropriate complementary feeding and nutrition-sensitive programs that address maternal and household socioeconomic factors, including female education and literacy (34, 35). At the same time, implementation of policies—even if they are set at macro levels—occurs at the local context.

In India, community health governance already exists with more than 500,000 Village Health Sanitation and Nutrition Committees (VHSNCs) and female community health workers (Accredited Social Health Activists, or ASHAs) (27). The VHSNC was established to empower local people and village councils to contribute to the governance of health and other public services (36). The VHSNCs are chaired by Gram Panchayat members at the village level and federated at higher levels. While the VHSNCs are expected to convene monthly meetings with community members, frontline health providers, and locally elected representatives to conduct local health planning and monitor health and nutrition services, many were found to be functioning poorly (27, 37, 38). However, the recently revised guidelines and an institutional support package from the Ministry of Health and Family Welfare provide important opportunities to improve VHSNC functions to fulfill its role in implementing equitable and efficient interventions. The local health workers (ASHAs) implement maternal and child nutrition services (39), and the Anganwadi centers provide basic health care activities, including nutrition education and supplementary care (36). Our village estimates of child anthropometric failures provide evidence to hold VHSNC and Gram Panchayat accountable for the optimal growth in children they serve. Moreover, village-level estimates of child anthropometric failures can inform local initiatives to be maximized in a more efficient and effective manner.

We recognize the potential measurement error resulting from the random displacement of GPS coordinates in the DHS clusters. While the clusters were restricted to be contained within the same district, they were randomly displaced by a maximum of 5 km. Our model attempts to correct the bias introduced by the small number of reliable cluster village mappings using the correction step, but it is unlikely that this approach can fully compensate for the unavailability of a relatively large number of labeled villages. Furthermore, this also complicates the process of getting uncertainty estimates for our predictions. Though, there appears to be no straightforward approach to compute CIs in our data setting. Fieldwork and data collection at village administrative units are necessary to validate our estimates further. Nevertheless, our study advances the field of global health research in two major directions. Methodologically speaking, our approach attempts to remedy the data limitations by a correction step to account for the fuzzy cluster to village mapping and exploits homogeneity in village attributes (via semisupervised learning) to compute predictions. At the same time, our approach does not lead to any increased risk of confidentiality breach of cluster identifiers in the DHS.

Our approach is different from the geospatial analysis of health indicators in Africa (12–15). Firstly, their unit of analysis is a cluster (survey and generated) that is expanded to a 5 × 5 km grid, leading to a final dataset of ∼50,000 clusters (12–15), which, in contrast to ours, is large enough to train regression models (a stacked ensemble) and validate using holdout sets. In India's context, this cluster/grid level analysis is potentially problematic in that it ignores the constitutional validity of village units. As such, analysis at the village level is more reasonable. The aggregation of covariates at the village level to a higher cluster level granularity presupposes homogeneity, which is not an appropriate assumption and is highly dependent on the constituent district and state in India.

Secondly, in the absence of data on population health and well-being covering all villages, this methodology can be adapted and applied to hundreds of indicators related to maternal and child nutrition and health available in the Indian DHS. With the routine collection of DHS, this methodology has far-reaching applicability to monitor progress in India. Additionally, our focus on village as a unit of analysis and target of inference can potentially shift the paradigm of policy discussion in India. Previously, developmental programs and policies concerning health and nutrition deliberated at state or district levels could not incorporate village variation due to lack of data. Our village estimates and ranking enable more informed prioritization and precise targeting such that the village administrations of the greatest need can play a more active role and be accountable for the health and well-being of the local population they intend to serve.

In conclusion, our analytical approach can be applied to understand the local distribution of diverse health, demographic, socioeconomic, and developmental status for which data are collected for only a subset of clusters with displaced GPS coordinates in India and other low- and middle-income countries. Attempts to provide rigorous assessment of the local burden of child anthropometric failure is an important step forward for precision public health policy making (2, 3, 40). While prior child nutrition policies and programs in India focused on districts for planning, implementation, and monitoring, we highlight that a majority of the geographic variation in child anthropometric failures occur at micro geographic levels of villages followed by macro administrative levels of states. The utility of fine-grained data can be fully leveraged when directed to specific local authorities who can translate them to action on the ground.

## Materials and Methods

### Data.

The first source of data we used to extract individual-level anthropometry measures was the DHS from 2016, also known as the National Family Health Survey, downloaded from https://dhsprogram.com/. The DHS collects data on health and family welfare issues from a representative sample of households to inform the Ministry of Health and Family Welfare and other agencies for policy and program purposes (41). The Indian DHS 2016 followed a stratified two-stage sample design. The 2011 Census served as the sampling frame for the selection of primary sampling units (PSUs), corresponding to villages in rural areas and census enumeration blocks in urban areas. Within every selected PSU, a complete household mapping and listing operation was conducted, and households were selected using systematic sampling with probability proportional to the size. The Indian DHS 2016, for the first time, covered all 640 districts across 36 states and union territories in India (41). In the children’s file, a total of 247,743 children aged less than 5 y were alive at the time of survey. We restricted our sample to those in rural areas (*n* = 188,521). After excluding 17,002 children (9.02%) who were missing height or weight measures, 171,519 children remained for the final analytical sample. This resulted in 19,882 clusters with data on children’s anthropometric measures across 627 districts and 36 states/union territories.

In the 2016 DHS for India, the GPS coordinates data on clusters were obtained via a special request. These survey cluster coordinates were collected in the field using GPS receivers, usually during the survey sample listing process. In general, the GPS readings for most clusters were accurate to less than 15 m. In order to ensure that respondent confidentiality was maintained, the GPS latitude/longitude positions were displaced for all clusters. The displacement was randomly carried out so that rural clusters contained a minimum of 0 and a maximum of 5 km of positional error. For 1% of the rural clusters, the displacement occurred up to 10 km. The displacement was restricted so that the points stay within the second administrative level of district (42). Of note, we use the term *clusters* to refer to PSUs from the DHS and the term *villages* to refer to villages from the Census to distinguish the source of data from which they are drawn from, but both represent equivalent units for substantive interpretation (i.e., DHS clusters are a small subsample of Census villages).

The second source of data were the 2011 Census village boundary and demographics data published by ML Infomap in 2016 (43). The data were accessed from the Harvard Geospatial Library by the Harvard Center for Geographic Analysis. ML Infomap collected individual taluka/tehsil paper maps and, where possible, the small-scale Census atlas maps from the Registrar General of India to scan and vectorize the boundaries of villages as polylines and the location of the village settlements as points (43). Then, geographic coordinates were sourced from high-resolution satellite images and transferred into the digitized maps by visibly identifying features such as roads, railways, or water that are common to both maps (43). The smallest geographic unit boundary for urban areas in the ML Infomap were towns, which are composed of multiple urban wards and are not comparable to villages in rural areas. In the Census of India 2011, an urban area was defined as the following: 1) all statutory places with a municipality, corporation, cantonment board, or notified town area committee, etc. or 2) a place with a minimum population of 5,000, at least 75% of a male working population engaged in nonagricultural pursuits, and a density of population of at least 400 per sq km (1,000 per sq mi). All other areas were classified as rural. All village boundaries were linked to the Census demographics by ML Infomap. A total of 654,153 units were offered as point locations of which 597,626 were inhabited rural villages. Additionally, the national base maps, which included international, state, district, and subdistrict boundaries, were acquired from ML Infomap.

The final source of data were the Census village amenities data from 2011 (22). The amenities data were acquired from the District Census Handbook on the website of the Office of the Registrar General and Census Commissioner, Government of India. Of the 640,948 villages that had amenities features, 597,618 were inhabited areas. Since the amenities features do not have geographical position for villages, whereas the demographic data from ML Infomap do, we linked these two datasets based on a 16-digit unique code derived from stringing identifiers for state, district, subdistrict, and village. This merge resulted in a total of 597,121 villages with geographical positions and complete Census data on demographic and amenities attributes, including sex ratio, proportion of workers, and presence of basic education, health, and infrastructure facilities. The complete list of demographic and amenities features used for our prediction modeling are presented in *SI Appendix*, Table S1.

### Child Anthropometric Failures.

We focused on three indicators of child anthropometric failures that are being monitored for the NNM and SDG targets: stunting (linear growth retardation reflecting cumulative growth deficits), wasting (a measure of body mass in relation to height or length that captures acute undernourishment), and underweight (a composite index accounting for both acute and chronic undernutrition) (20). In the 2016 DHS for India, a child’s weight was measured by trained health investigators using digital solar-powered scales along with adjustable Shorr measuring boards (41). Standing height was obtained for children older than 24 mo, and recumbent length was measured with children lying on the board placed on a flat surface for children younger than 24 mo (41). The raw height and weight measures were transformed into age- and sex-specific *z*-scores based on the World Health Organization child growth reference standards to construct binary outcomes of stunting defined as height-for-age *z*-scores <−2 SD, underweight defined as weight-for-age *z*-scores <−2 SD, and wasting defined as weight-for-height *z*-scores <−2 SD (44).

### Analysis.

We first used DHS data to estimate cluster-specific predicted probabilities of child stunting, wasting, and underweight. We produced precision-weighted estimations based on hierarchical logistic regression modeling to account for the complex survey design and sampling variability (45–47). There are several advantages to using this statistical modeling for small area estimation. All the information in the data is pooled to borrow strength such that poorly estimated cluster-specific predictions can benefit from the information for other clusters (45–47). That is, unreliable cluster-specific fixed estimates are differentially shrunken or smoothed toward the overall mean, which is based on all the data and hence generate more appropriately conservative estimates (45–47). We specified a four-level logistic regression model with child *i* (level one) nested within cluster *j* (level two), district *k* (level three), and state *l* (level four) for each outcome $logit\left(\pi_{ijkl}\right)=\beta +\left(u_{jkl}+v_{kl}+f_{l}\right)$, where the term $u_{jkl}$ denotes cluster-specific residuals with a variance of $\sigma_{u}^{2}$ assuming $u_{jkl}∼N\left(0,\sigma_{u}^{2}\right)$, $v_{kl}$ denotes district-specific residuals with a variance of $\sigma_{v}^{2}$ assuming $v_{kl}∼N\left(0,\sigma_{v}^{2}\right)$, and $f_{l}$ denotes state-specific residuals with a variance of $\sigma_{f}^{2}$ assuming $f_{l}∼N\left(0,\sigma_{f}^{2}\right)$. For binary outcome models, the variance at the individual level is approximated using a latent variable method as $\pi^{2}/3$ (48). The cluster-specific predicted logit values were converted to probabilities by taking the average over the simulations, that is, $\exp\left(\beta +\left(u_{jkl}+v_{kl}+f_{l}\right)\right)/\left(1+\exp\left(\beta +\left(u_{jkl}+v_{kl}+f_{l}\right)\right)\right)$. Multilevel modeling was performed in the MLwiN 3.00 software program via Markov chain Monte Carlo methods using a Gibbs sampler with default prior distributions of iterative generalized least squares estimations as starting values, a burn-in of 500 cycles, and monitoring of 5,000 iterations of chains (49). The chains of the loading estimates for all parameters were checked for convergence (49).

The precision-weighted child anthropometric failure estimates for 19,882 labeled clusters were linked to villages using the randomly displaced GPS coordinates data from DHS and ML Infomap shapefiles. The Census covers all Indian villages, whereas the DHS data includes a small subsample of villages (clusters) and, thus, are spatially isolated. In order to link DHS clusters to Census villages, we performed the following five steps. First, spatial boundaries for villages were generated for six states of Andaman and Nicobar Island, Arunachal Pradesh, Manipur, Meghalaya, Mizoram, and Nagaland because they were offered as point locations without boundaries in ML Infomap. For these “point only” village locations, we used the ArcGIS create Thiessen polygons function to represent village boundaries. More specifically, we selected villages per district, produced the Thiessen polygons, and clipped the result by the district boundaries. As a result, this produced polygon boundaries for all villages. Second, we generated 5 km buffers for DHS survey cluster points to account for the random displacement applied to GPS coordinates. Third, we selected clusters and villages in each state, and then linked clusters’ buffers with villages by spatial overlay (ArcGIS identify). From this result, we selected village/cluster matches where both the Census village and survey clusters were classified as “rural” and contained the same district identifier. This procedure ensured that Census villages and survey clusters share the same characteristics. Fourth, these filtered results were dissolved by deleting the duplicated results, leaving 623,463 pairs of possible cluster-to-village matches. Fifth, the results were further filtered by population. Since individuals were subsampled from clusters in DHS, it is not possible for the population within a cluster to exceed the population within a matching village. Accordingly, we restricted the cluster-to-villages pairs to those where village population was larger than the cluster population. Of the 19,882 labeled clusters, 122 had an erroneous reported latitude/longitude of 0,0, and 265 clusters were dropped because of filtering by character (i.e., the only cluster within 5 km of village was in a different district) and population (i.e., villages had a smaller population than the corresponding cluster). In the final linked dataset, we had 551,348 cluster-to-village pairs. The maximum number of villages matched to a single cluster was 171, and the maximum number of clusters matched to a village was 22. In this process we maintain the integrity of the data source and the confidentiality of cluster identifiers from DHS.

As discussed before, the main issue confronting a country wide prediction model for the health indicators is the absence of well-defined labeled data. The many-to-many possible mapping of the villages to the clusters and the consequent uncertainty of child anthropometric failure estimates prevented us from adopting a standard machine learning prediction model. There were 19,882 clusters that could potentially be associated with 339,072 villages and 286,051 villages that were not mapped to any cluster. Out of the 19,882 clusters, only 258 had a single village mapped to it. In case the same village is mapped to more than one cluster, we average the estimate across these clusters (thereby reducing uncertainty, *SI Appendix*, Fig. S1), finally obtaining 78 villages with one-to-one cluster mapping. We refer to this set as the reliable label set. Given the small number of reliable labels and no clear-cut way to exploit the much larger set of 19,624 fuzzy labeled clusters, the data as such do not conform to standard supervised regression models. Even if we could somehow assign this fuzzy set to villages, the overwhelming majority of villages remain unlabeled. Hence, it is unlikely that using such a small set of training data to generalize predictions to this majority would produce estimates that captured the true distribution.

Therefore, we proposed an extension of a semisupervised model (50) that accommodates the limitation of the data by using the following two steps:

• Initialization—produce semisupervised estimates for each of the stunting, underweight, and wasting indicators using an initial labeled set.
• Correction—update the 19,624 fuzzy labels based on the proximity to known cluster estimates and execute semisupervised regression to obtain updated predictions.

In the first step, we produce an estimate of the predictions using initial labels. We considered the 78 villages corresponding to clusters that are mapped to one village only. We then use all the remaining unlabeled ones in a semisupervised regression model for each of the indicators (see next section). The first set of predictions is expected to be biased, as it relies on a small-labeled set. To remedy this, we added a correction step that updates the labels for fuzzy clusters. For each fuzzy cluster, the village among its possible linking candidate villages with the smallest error (in absolute value) compared to the preliminary prediction. Using this approach, we updated our labeled training data to get more accurate estimates to reuse in the semisupervised regression framework. These two steps can then be generalized in an iterative procedure as discussed in the next section.

### Semisupervised Learning Framework.

For the notation, let Х be the design matrix corresponding to the d Census amenities and demographic features for a total of n villages and Y_S_, Y_U_, and Y_W_ be the vectors corresponding to known (m = 78 in our analysis) and unknown health indicators (n-m) (stunting, underweight, and wasting). Also, let C be the set of 19,624 clusters of villages with known stunting, underweight, and wasting estimates, and let $V_{c}$ be the set of villages that map to cluster c∈C .

In the general semisupervised framework, we are given labeled pairs (samples with known output values) $\left(x_{1},y_{1}\right),\;\left(x_{2},\;y_{2}\right),\ldots ,\;\left(x_{l},\;y_{l}\right)$ and unlabeled points $x_{l+1},\ldots ,\;x_{l+u}$, and the goal is to estimate a mapping f:X→Y between the input and the output using both labeled and unlabeled data. There are different ways of achieving this goal based on discriminative or graph-based approaches (50). The central idea of semisupervised learning is that unlabeled data can often help in better estimation of the underlying mapping than by just using the labeled data alone. A simple example is shown in *SI Appendix*, Fig. S2. It becomes clear that if unlabeled data are used additionally, the separating boundary is different compared to the one obtained using only labeled data.

Our approach is flexible in the choice of regression model used. We chose to work with semisupervised models because of our belief that attribute-level homogeneity is an important factor in getting interpretable estimates. Specifically, we used the framework of joint harmonic functions (51). The method essentially combines two assumptions behind commonly used semisupervised approaches, namely the cluster and the harmonic approximation assumption. The cluster assumption captures the intuition that villages similar in attributes (based on some proximity metric) have similar health indicators, and the harmonic assumption states that the indicator for a village can be approximated by a weighted average of its neighbors in a proximity graph. A detailed description of the method is beyond the scope of this paper, and we refer the reader to joint harmonic functions (51).

In the next section, we describe an approach that builds upon any semisupervised regression method to get the unknown stunting, underweight, and wasting estimates for all the villages.

### Bias-Corrected Semisupervised Regression.

Box 1. Algorithm for bias-corrected semisupervised regression

• **Input**
  • $Y^{0}\in R^{n}$—initial vector of known (**m)** and unknown (**n-m**) health indicator (S, U, or W), **Х**—matrix of features for all villages, and $Y_{C}$—vector of known health indicators for **|C|** clusters. Set the unknown values in **Y**^**0**^ to **0** and $L^{0}$ be the set of **m**-labeled villages.
• **Initialization**
  • **Y**^**1**^ **= Semi-Supervised** (**Y**^**0**^**, Х,** $L^{0}$)
• **Correction**
  1. For each labeled cluster ***c*** and the set villages mapped to it (***V***_***c***_), find the village with the smallest gap between its predicted value and the indicator associated with the cluster and set.
    a. $L^{1}=\left\{\;arg\underset{j}{\min}\;\left|Y_{j}^{1}-Y_{V}{}_{c}\right|\;:\forall c,\;1\leq c\leq \left|C\right|\;,\;j\in V_{c}\right\}$
  2. Revert the estimates for the **m** villages in $L^{1}$ to $L^{0}$**.**
  3. **Y**^**out**^ **= Semi-Supervised** (**Y**^**1**^**, Х,** $L^{1}$)
• **Output**
• Return **Y**^**out**^

The algorithm begins with the m-labeled villages and produces a first set of predictions based on any semisupervised regression approach and then updates the labeled set to m+|C| by comparing the predictions to the set of available cluster level indicators (Box 1). This is done by choosing a village in each of the clusters that has the closest predicted value (from the initialization) to the indicator estimate from the cluster. This method is run separately for each of the health indicators. An iterative version of the method is shown below where we repeat the steps one to three of the correction method (Box 2).

Box 2. An iterative version of the method for bias correction

• **Input**
  o **Y**^**0**^—initial vector of known (**m)** and unknown (**n-m**) health indicator (S, U, or W), **Х**—matrix of features for all villages, and $Y_{C}$—vector of known health indicators for **|C|** clusters; number of iterations **T**.
• Set **t = 1,** the unknown values in **Y**^**1**^ to **0**, and let $L^{0}$ be the set of **m**-labeled villages
• WHILE (t≤T)
  1. **Y**^**t**^ **= Semi-Supervised** (**Y**^**t-1**^, **Х,** $L^{t-1}$)
  2. For each labeled cluster and the villages mapped to it (***V***_***c***_), find the village with the smallest gap between its predicted value and the indicator associated with the cluster and set.
    a. $L^{t}=\left\{\;arg\underset{j}{\min}\;\left|Y_{j}^{t}-Y_{V}{}_{c}\right|\;:\forall c,\;1\leq c\leq \left|C\right|\;,\;j\in V_{c}\right\}$
  3. Revert the estimates for the **m** villages in $L^{t}$ to $L^{1}$**.**
END
• Return **Y**^**T**^**—**set of predictions for all **n** villages.

## Sensitivity Analysis

We performed three sensitivity analyses of our method: first, by checking the robustness of our method to a randomized initial labeling method; second, by checking intracluster variation across clusters of varying sizes; and third, by comparing the district-level summary of stunting, underweight, and wasting from the predicted village estimates and the labeled clusters. For the first analysis, instead of assigning labels based on one-to-one village cluster mapping, we randomly assigned villages to cluster health indicators. We plot the histograms and show the first three quartiles (25th, 50th, and 75th percentile) for each random assignment-based predictions (from iteration number five of the method) in *SI Appendix*, Fig. S3. For stunting, underweight, and wasting, it is evident that the predictions are robust to the assignment. The second analysis aimed to assess whether clusters that have a smaller number of possible matching villages also have a range of predicted values that is tighter around the precision-weighted estimates. In *SI Appendix*, Fig. S4**,** we plot the range of predictions across clusters of increasing sizes. Intuitively, we expect that the clusters with a larger number of matching villages will have a higher range. We do indeed observe a growing trend in the range as the number of mapped villages increase. Lastly, the correlation for district-wide mean between the predicted village estimates versus the labeled clusters was the strongest for underweight (*r* = 0.87) followed by stunting (*r* = 0.75) and wasting (*r* = 0.66).

## Supplementary Material

Supplementary File

Supplementary File

Supplementary File

## Data Availability

The data on the 2015/2016 Indian DHS are available from https://dhsprogram.com/data/dataset/India_Standard-DHS_2006.cfm?flag=1. The data on GPS coordinates for the Indian DHS survey clusters are available only via special request. The 2011 Census village boundary and demographics data were purchased from ML Infomap, and are available from https://www.mlinfomap.com/map-data.php. The 2011 Census village amenities data are publicly available from the Office of the Registrar General and Census Commissioner, Government of India, https://censusindia.gov.in/2011census/dchb/DCHB.html. All data generated from the prediction modeling are included in Dataset S1. Analytic codes used for prediction modeling are provided as Dataset S2. All other data are included in the article and/or supporting information.
