# Supplementary material for: Precision mapping child undernutrition for nearly 600,000 inhabited census villages in India
Source: Proc Natl Acad Sci U S A. 2021 Apr 26;118(18):e2025865118. doi: 10.1073/pnas.2025865118 (PMC8106321; doi:10.1073/pnas.2025865118)
Supplement: Supplementary File [file pnas.2025865118.sapp.pdf]

Supplementary Information for
Precision mapping child undernutrition for nearly 600,000 inhabited census villages in India

Rockli Kim, Avleen Bijral, Yun Xu, Xiuyuan Zhang, Jeffrey C. Blossom, Akshay Swaminathan, Gary King, Alok Kumar, Rakesh Sarwal, Juan M. Lavista Ferres, S.V. Subramanian

Correspondence to S.V. Subramanian
Email: svsubram@hsph.harvard.edu

This PDF file includes:

Figures S1 to S4
Tables S1
Legend for Dataset S1 to S2

Other supplementary materials for this manuscript include the following:

Datasets S1 to S2

Fig S1. The initial values for villages are obtained by taking the average of the estimates corresponding to the clusters these villages are mapped to

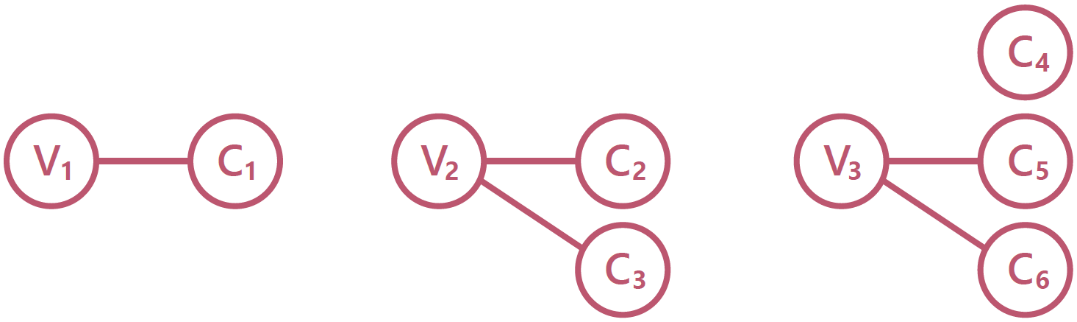

Fig S2. 1-dimensional illustration of how unlabeled data can help in a simple classification problem

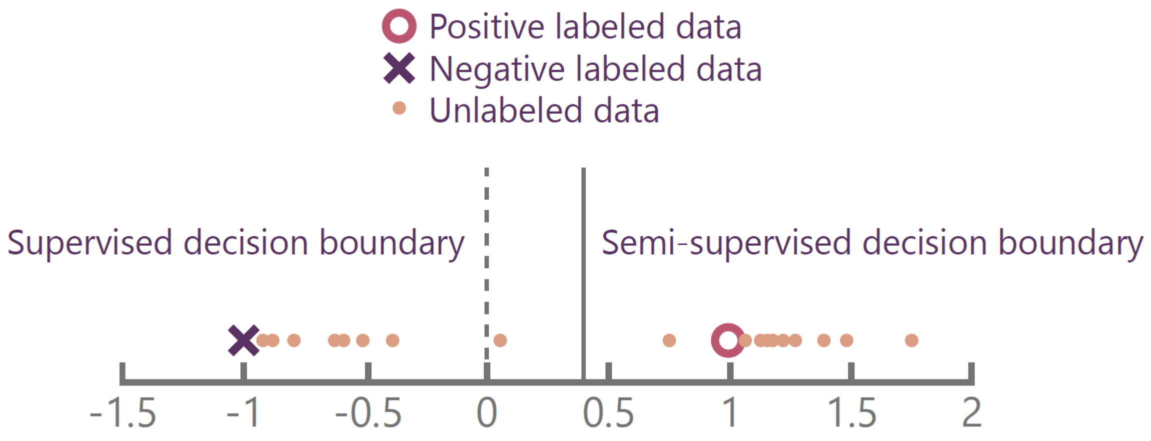

Fig S3. Prediction histograms for 20 randomized initial label assignments for (a) stunting, (b) underweight, and (c) wasting

(a) Stunting

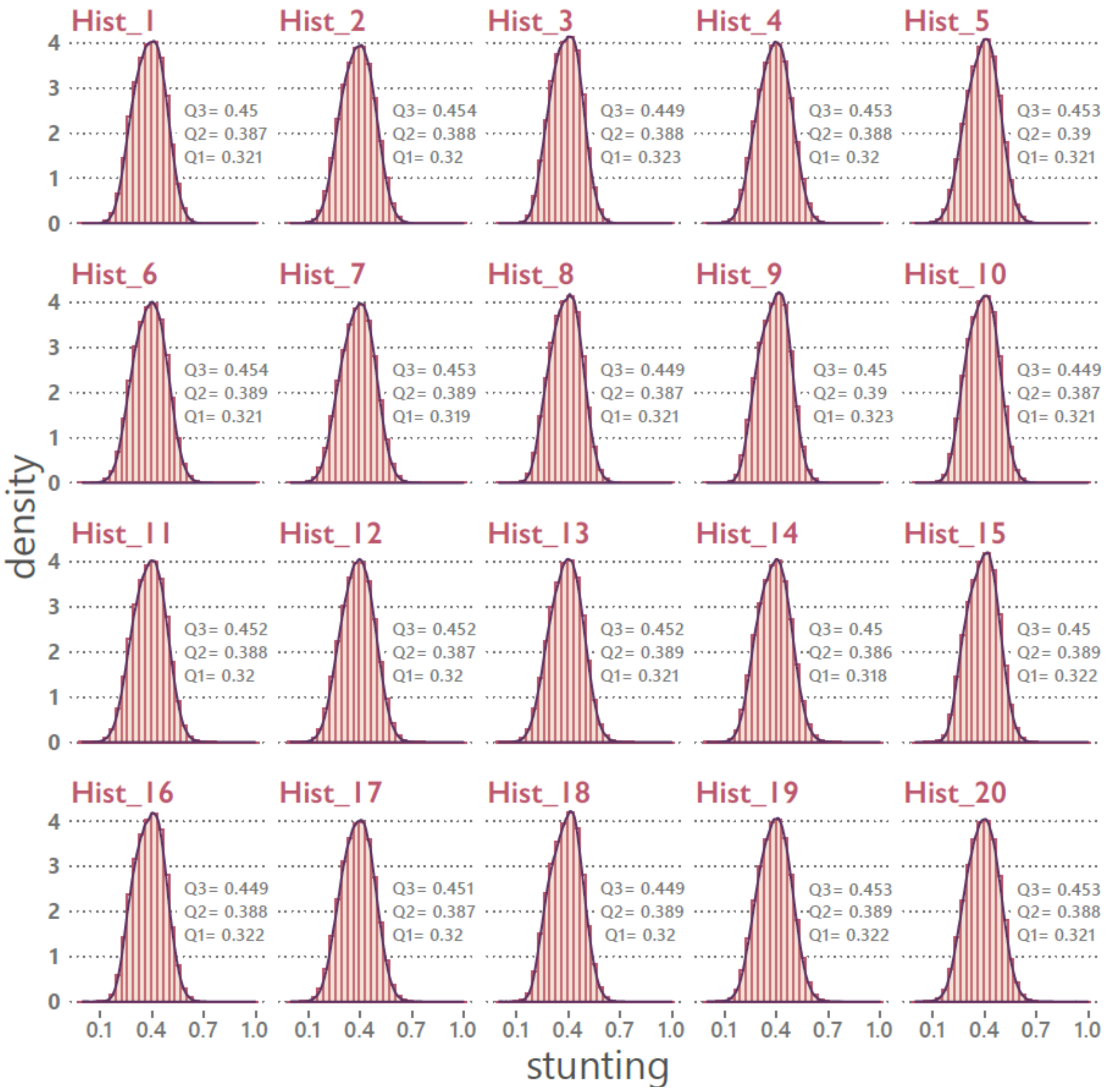

(b) Underweight

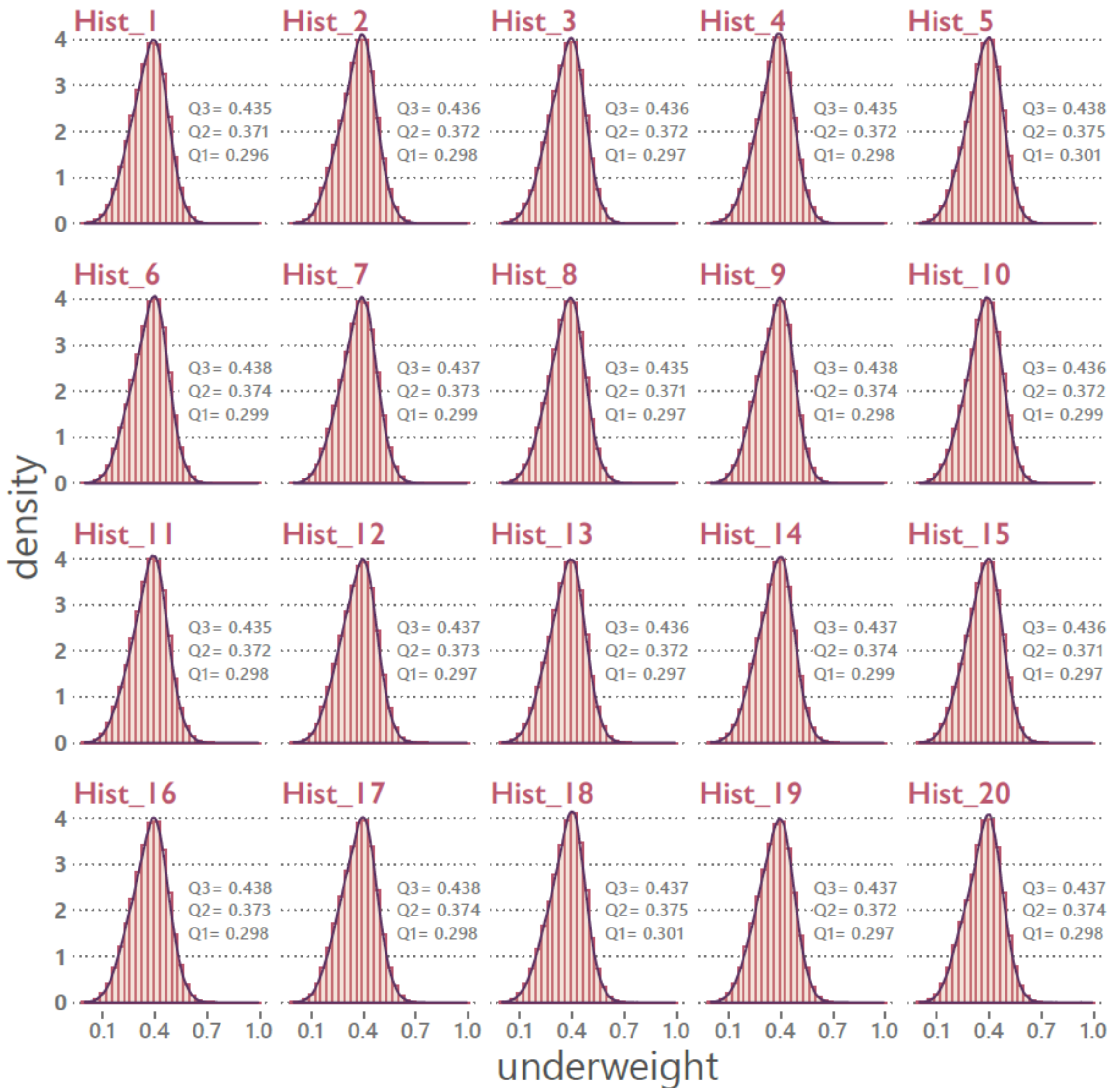

(c) Wasting

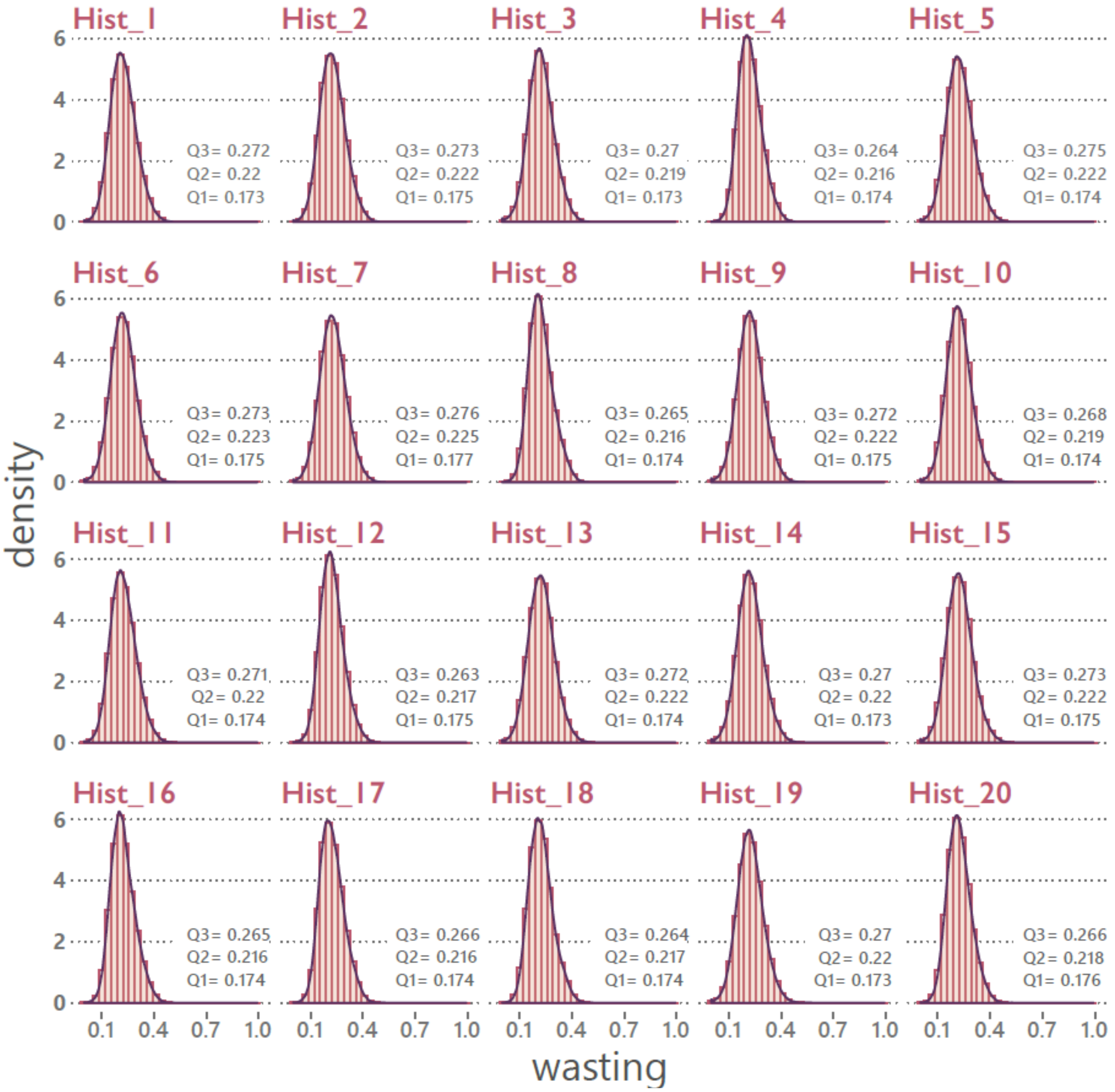

Hist_1
Q3= 0.272
Q2= 0.22
Q1= 0.173
Hist_2
Q3= 0.273
Q2= 0.222
Q1= 0.175
Hist_3
Q3= 0.27
Q2= 0.219
Q1= 0.173
Hist_4
Q3= 0.264
Q2= 0.216
Q1= 0.174
Hist_5
Q3= 0.275
Q2= 0.222
Q1= 0.174
Hist_6
Q3= 0.273
Q2= 0.223
Q1= 0.175
Hist_7
Q3= 0.276
Q2= 0.225
Q1= 0.177
Hist_8
Q3= 0.265
Q2= 0.216
Q1= 0.174
Hist_9
Q3= 0.272
Q2= 0.222
Q1= 0.175
Hist_10
Q3= 0.268
Q2= 0.219
Q1= 0.174
Hist_11
Q3= 0.271
Q2= 0.22
Q1= 0.174
Hist_12
Q3= 0.263
Q2= 0.217
Q1= 0.175
Hist_13
Q3= 0.272
Q2= 0.222
Q1= 0.174
Hist_14
Q3= 0.27
Q2= 0.22
Q1= 0.173
Hist_15
Q3= 0.273
Q2= 0.222
Q1= 0.175
Hist_16
Q3= 0.265
Q2= 0.216
Q1= 0.174
Hist_17
Q3= 0.266
Q2= 0.216
Q1= 0.174
Hist_18
Q3= 0.264
Q2= 0.217
Q1= 0.174
Hist_19
Q3= 0.27
Q2= 0.22
Q1= 0.173
Hist_20
Q3= 0.266
Q2= 0.218
Q1= 0.176
density
wasting
0
2
4
6
0.1
0.4
0.7
1.0

Fig S4. Range variation in (a) stunting, (b) underweight, and (c) wasting as the number of mapped villages increases.

(a) Stunting

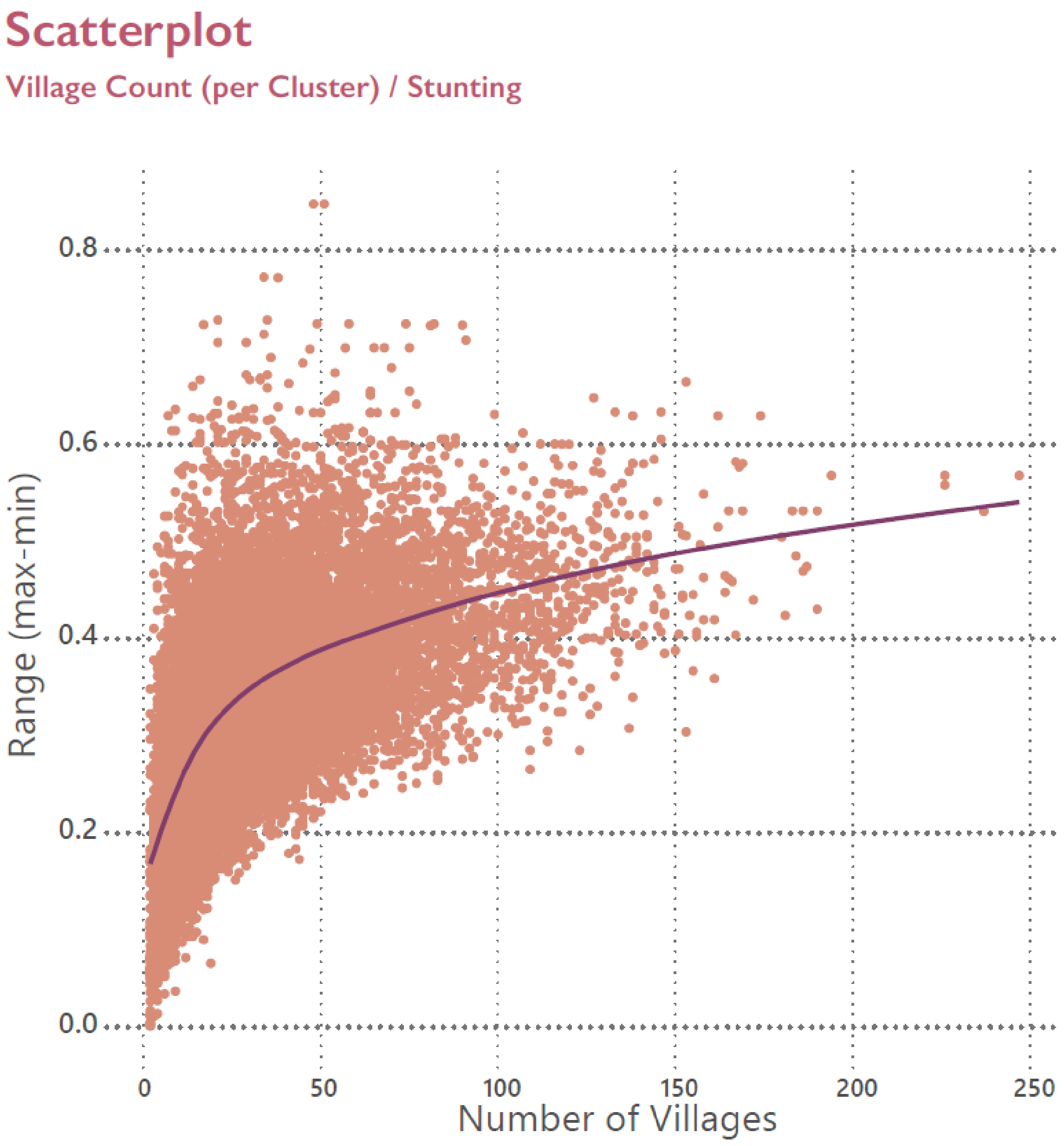

(b) Underweight

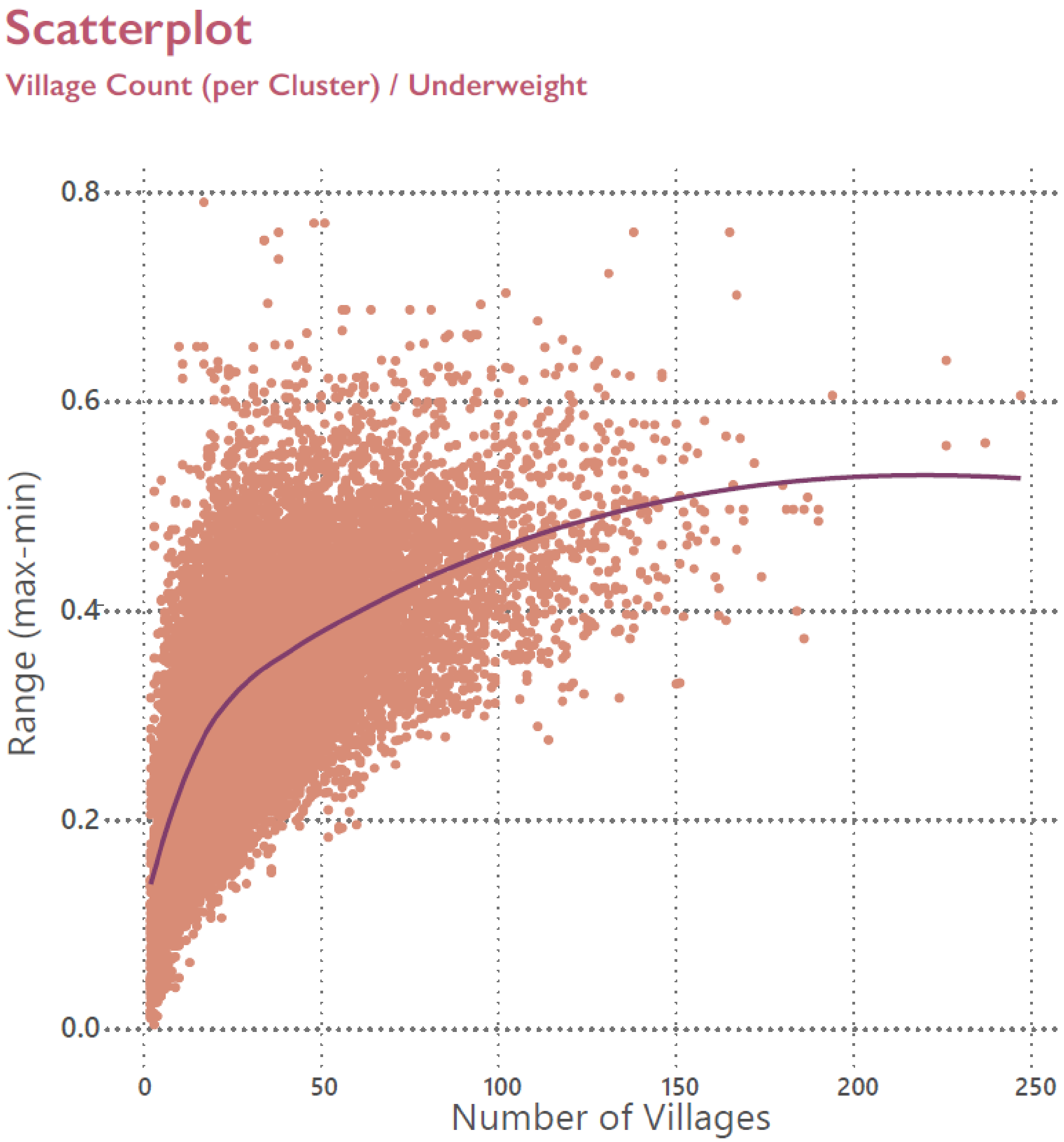

(c) Wasting

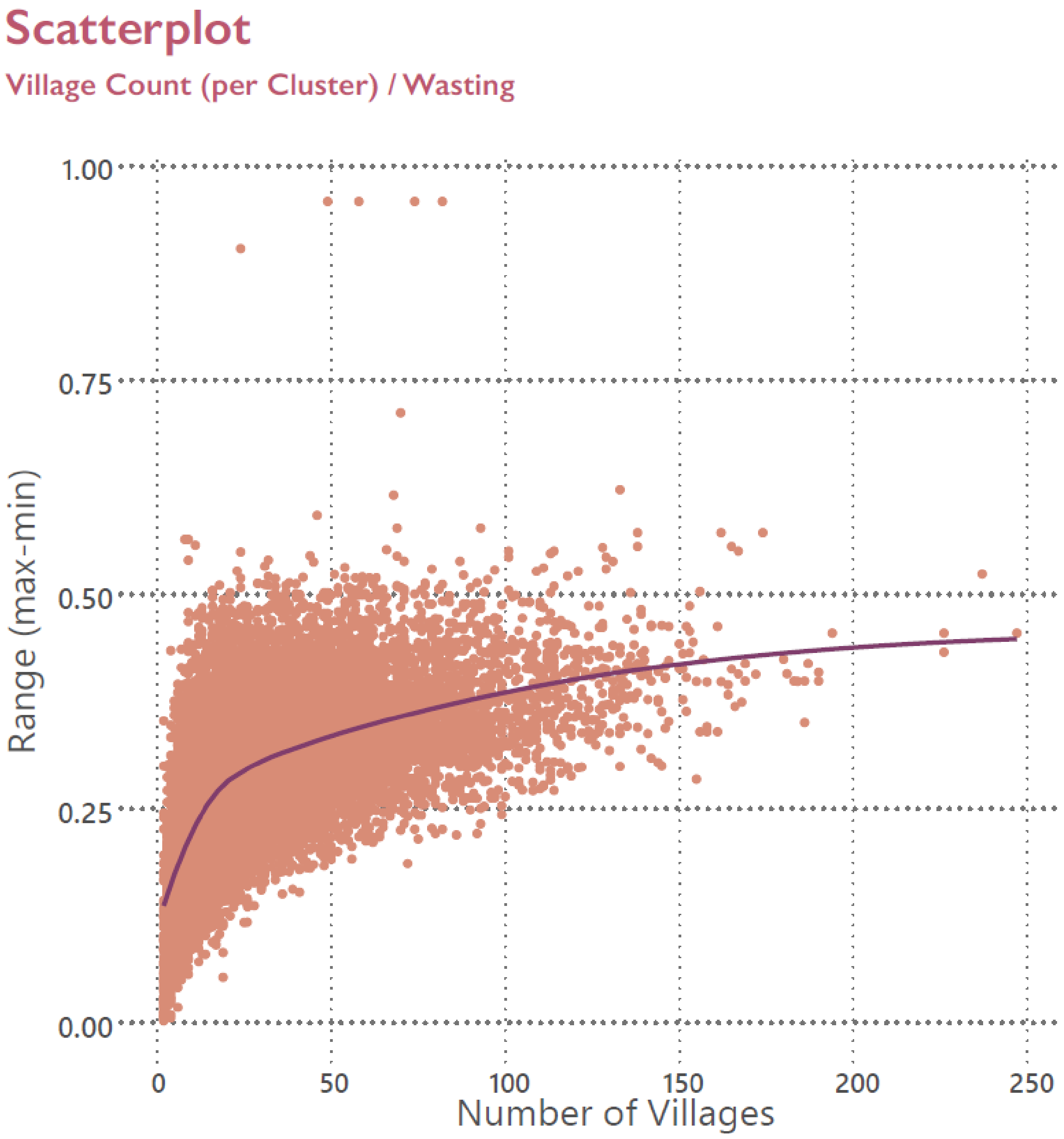

Scatterplot
Village Count (per Cluster) / Wasting
1.00
0.75
0.50
0.25
0.00
Range (max-min)
0
50
100
150
200
250
Number of Villages

Table S1. List of 2011 Census demographic and amenities features used for prediction models

Variable name	Definition	Note
tot_hh	Total number of household	Raw variable from ML Infomap demographic file
m_mnw	Male Main Workers	Raw variable from ML Infomap demographic file
f_mnw	Female Main Workers	Raw variable from ML Infomap demographic file
m_cult	Male Cultivators	Raw variable from ML Infomap demographic file
f_cult	Female Cultivators	Raw variable from ML Infomap demographic file
m_aglb	Male Agricultural Labourers	Raw variable from ML Infomap demographic file
f_aglb	Female Agricultural Labourers	Raw variable from ML Infomap demographic file
m_mfhh	Male Household industry workers	Raw variable from ML Infomap demographic file
f_mfhh	Female Household industry workers	Raw variable from ML Infomap demographic file
m_oth_w	Male other workers	Raw variable from ML Infomap demographic file
f_oth_w	Female other workers	Raw variable from ML Infomap demographic file
m_mrg_3_6	Male Marginal Workers- worked for 3 months or more but less than 6 months	Raw variable from ML Infomap demographic file
f_mrg_3_6	Female Marginal Workers- worked for 3 months or more but less than 6 months	Raw variable from ML Infomap demographic file
m_m_cl_3_6	Male Marginal Cultivators- worked for 3 months or more but less than 6 months	Raw variable from ML Infomap demographic file
f_m_cl_3_6	Female Marginal Cultivators- worked for 3 months or more but less than 6 months	Raw variable from ML Infomap demographic file
m_mag_3_6	Male Marginal Agricultural Labourers- worked for 3 months or more but less than 6 months	Raw variable from ML Infomap demographic file
f_mag_3_6	Female Marginal Agricultural Labourers- worked for 3 months or more but less than 6 months	Raw variable from ML Infomap demographic file
m_mhh_3_6	Male Marginal Workers in Household Industries- worked for 3 months or more but less than 6 months	Raw variable from ML Infomap demographic file
f_mhh_3_6	Female Marginal Workers in Household Industries- worked for 3 months or more but less than 6 months	Raw variable from ML Infomap demographic file
m_mot_3_6	Male Marginal Other Workers- worked for 3 months or more but less than 6 months	Raw variable from ML Infomap demographic file

f_mot_3_6	Female Marginal Other Workers- worked for 3 months or more but less than 6 months	Raw variable from ML Infomap demographic file
m_mrg_0_3	Male Marginal Workers- worked for less than 3 months	Raw variable from ML Infomap demographic file
f_mrg_0_3	Female Marginal Workers- worked for less than 3 months	Raw variable from ML Infomap demographic file
m_m_cl_0_3	Male Marginal Cultivators- worked for less than 3 months	Raw variable from ML Infomap demographic file
f_m_cl_0_3	Female Marginal Cultivators- worked for less than 3 months	Raw variable from ML Infomap demographic file
m_mag_0_3	Male Marginal Agricultural Labourers- worked for less than 3 months	Raw variable from ML Infomap demographic file
f_mag_0_3	Female Marginal Agricultural Labourers- worked for less than 3 months	Raw variable from ML Infomap demographic file
m_mhh_0_3	Male Marginal Workers in Household Industries- worked for less than 3 months	Raw variable from ML Infomap demographic file
f_mhh_0_3	Female Marginal Workers in Household Industries- worked for less than 3 months	Raw variable from ML Infomap demographic file
m_mot_0_3	Male Marginal Other Workers	Raw variable from ML Infomap demographic file
f_mot_0_3	Female Marginal Other Workers	Raw variable from ML Infomap demographic file
m_nnw	Male Non-workers	Raw variable from ML Infomap demographic file
f_nnw	Female Non-workers	Raw variable from ML Infomap demographic file
population_per_hectare	tot_pop/Total_Geographical_Area__in_Hect	Derived variable from ML Infomap demographic file
sex_ratio	f_pop/ m_pop	Derived variable from ML Infomap demographic file
child_population	tot_l6/tot_pop	Derived variable from ML Infomap demographic file
child_sex_ratio	f_l6 / m_l6	Derived variable from ML Infomap demographic file
proportion_SC	tot_sc / tot_pop	Derived variable from ML Infomap demographic file
proportion_ST	tot_st / tot_pop	Derived variable from ML Infomap demographic file
proportion_total_literate	tot_lit / (tot_pop - tot_l6)	Derived variable from ML Infomap demographic file
proportion_male_literate	m_lit / (m_pop - m_l6)	Derived variable from ML Infomap demographic file
proportion_female_literate	f_lit / (f_pop - f_l6)	Derived variable from ML Infomap demographic file
proportion_workers	tot_w / (tot_pop - tot_l6)	Derived variable from ML Infomap demographic file
proportion_male_workers	m_w / (m_pop - m_l6)	Derived variable from ML Infomap demographic file
proportion_female_workers	f_w / (f_pop - f_l6)	Derived variable from ML Infomap demographic file
proportion_AGLB	tot_aglb / tot_w	Derived variable from ML Infomap demographic file

proportion_MRW	tot_mrw / tot_w	Derived variable from ML Infomap demographic file
proportion_MRG_3_6	t_mrg_3_6 / tot_w	Derived variable from ML Infomap demographic file
proportion_MRG_0_3	t_mrg_0_3 / tot_w	Derived variable from ML Infomap demographic file
District_Head_Quarter___Distance	District Head Quarter (Distance in km)	Raw variable from village amenities file
Nearest_Statutory_Town__Distance	Nearest Statutory Town (Distance in km)	Raw variable from village amenities file
Total_Geographical_Area__in_Hect	Total Geographical Area (in Hectares)	Raw variable from village amenities file
Govt_Pre_Primary_School__Nursery	Govt Pre-Primary School (Nursery/LKG/UKG) (Status A(1)/NA(2))	Derived variable from village amenities file
Private_Pre___Primary_School__Nu	Private Pre - Primary School (Nursery/LKG/UKG) (Status A(1)/NA(2))	Derived variable from village amenities file
Govt_Primary_School__Status	Govt Primary School (Status A(1)/NA(2))	Derived variable from village amenities file
Private_Primary_School__Status_A	Private Primary School (Status A(1)/NA(2))	Derived variable from village amenities file
Govt__Middle_School__Status	Govt Middle School (Status A(1)/NA(2))	Derived variable from village amenities file
Private_Middle__School__Status_A	Private Middle School (Status A(1)/NA(2))	Derived variable from village amenities file
Govt_Secondary_School__Status_A_	Govt Secondary School (Status A(1)/NA(2))	Derived variable from village amenities file
Private_Secondary_School__Status	Private Secondary School (Status A(1)/NA(2))	Derived variable from village amenities file
Govt_Senior_Secondary_School__St	Govt Senior Secondary School (Status A(1)/NA(2))	Derived variable from village amenities file
Private_Senior_Secondary_School	Private Senior Secondary School (Status A(1)/NA(2))	Derived variable from village amenities file
Govt__Arts_and_Science_Degree_Co	Govt Arts and Science Degree College (Status A(1)/NA(2))	Derived variable from village amenities file
Private__Arts_and_Science_Degree	Private Arts and Science Degree College (Status A(1)/NA(2))	Derived variable from village amenities file
Govt_Engineering_College__Status	Govt Engineering College (Status A(1)/NA(2))	Derived variable from village amenities file
Private_Engineering_College__Sta	Private Engineering College (Status A(1)/NA(2))	Derived variable from village amenities file
Govt_Medicine_College__Status_A_	Govt Medicine College (Status A(1)/NA(2))	Derived variable from village amenities file
Private_Medicine__College__Statu	Private Medicine College (Status A(1)/NA(2))	Derived variable from village amenities file
Govt_Management_Institute__Statu	Govt Management Institute (Status A(1)/NA(2))	Derived variable from village amenities file
Private_Management_Institute__St	Private Management Institute (Status A(1)/NA(2))	Derived variable from village amenities file
Govt_Polytechnic_Status	Govt Polytechnic (Status A(1)/NA(2))	Derived variable from village amenities file
Private_Polytechnic_Status	Private Polytechnic (Status A(1)/NA(2))	Derived variable from village amenities file
Govt_Vocational_Training_School_	Govt Vocational Training School/ITI (Status A(1)/NA(2))	Derived variable from village amenities file

Private_Vocational_Training_Scho	Private Vocational Training School/ITI (Status A(1)/NA(2))	Derived variable from village amenities file
Government_Non_Formal_Training_C	Government Non Formal Training Centre (Status A(1)/NA(2))	Derived variable from village amenities file
Private_Non_Formal_Training_Cent	Private Non Formal Training Centre (Status A(1)/NA(2))	Derived variable from village amenities file
Government_School_For_Disabled__	Government School For Disabled (Status A(1)/NA(2))	Derived variable from village amenities file
Private_School_For_Disabled___St	Private School For Disabled (Status A(1)/NA(2))	Derived variable from village amenities file
Government_Others_Status	Government Others (Status A(1)/NA(2))	Derived variable from village amenities file
Private_Others_Status	Private Others (Status A(1)/NA(2))	Derived variable from village amenities file
Community_Health_Centre_Status	Community_Health_Centre (Status A(1)/NA(2))	Derived variable from village amenities file
Primary_Health_Centre_Status	Primary_Health_Centre (Status A(1)/NA(2))	Derived variable from village amenities file
Primary_Health_Sub_Centre_Status	Primary Health Sub Centre (Status A(1)/NA(2))	Derived variable from village amenities file
Maternity_Child_Center_Status	Maternity And Child Welfare Centre(Status A(1)/NA(2))	Derived variable from village amenities file
TB_Clinic_Status	TB Clinic(Status A(1)/NA(2))	Derived variable from village amenities file
Hospital_Allopathic_Status	Hospital Allopathic(Status A(1)/NA(2))	Derived variable from village amenities file
Hospital_Alternative_Status	Hospital Alternative Medicine(Status A(1)/NA(2))	Derived variable from village amenities file
Dispensary_Status	Dispensary(Status A(1)/NA(2))	Derived variable from village amenities file
Veterinary_Hospital_Status	Veterinary Hospital(Status A(1)/NA(2))	Derived variable from village amenities file
Mobile_Health_Clinic_Status	Mobile Health Clinic(Status A(1)/NA(2))	Derived variable from village amenities file
Family_Welfare_Center_Status	Family Welfare Centre(Status A(1)/NA(2))	Derived variable from village amenities file
Non_Gov_Medical_Out_Status	Non Government Medical facilities Out Patient(Status A(1)/NA(2))	Derived variable from village amenities file
Non_Gov_Medical_InOut_Status	Non Government Medical facilities In and Out Patient(Status A(1)/NA(2))	Derived variable from village amenities file
Non_Gov_Medical_Char_Status	Non Government Medical facilities Charitable(Status A(1)/NA(2))	Derived variable from village amenities file
Non_Gov_Medical_MBBS_Status	Non Government Medical facilities Medical Practitioner with MBBS Degree(Status A(1)/NA(2))	Derived variable from village amenities file
Non_Gov_Medical_OtherDeg_Status	Non Government Medical facilities Medical Practitioner with Other Degree(Status A(1)/NA(2))	Derived variable from village amenities file
Non_Gov_Medical_NoDeg_Status	Non Government Medical facilities Medical Practitioner with No Degree(Status A(1)/NA(2))	Derived variable from village amenities file

Non_Gov_Medical_Faith_Status	Non Government Medical facilities Traditional Pracitioner and Faith Healer(Status A(1)/NA(2))	Derived variable from village amenities file
Non_Gov_Medical_Shop_Status	Non Government Medical facilities Medicine Shop(Status A(1)/NA(2))	Derived variable from village amenities file
Non_Gov_Medical_Others_Status	Non Government Medical facilities Other(Status A(1)/NA(2))	Derived variable from village amenities file
Tap_Water_Treated_Status	Tap Water-Treated (Status A(1)/NA(2))	Derived variable from village amenities file
Tap_Water_Untreated_Status	Tap Water Untreated (Status A(1)/NA(2))	Derived variable from village amenities file
Covered_Well_Status	Covered Well (Status A(1)/NA(2))	Derived variable from village amenities file
Uncovered__Well_Status	Uncovered Well (Status A(1)/NA(2))	Derived variable from village amenities file
Hand_Pump_Functioning_Status	Hand Pump (Status A(1)/NA(2))	Derived variable from village amenities file
Tube_Wells_Borehole_Status	Tube Wells/Borehole (Status A(1)/NA(2))	Derived variable from village amenities file
Spring_Functioning_Status	Spring (Status A(1)/NA(2))	Derived variable from village amenities file
River_Canal_Functioning_Status	River/Canal (Status A(1)/NA(2))	Derived variable from village amenities file
Tank_Pond_Lake_Status	Tank/Pond/Lake (Status A(1)/NA(2))	Derived variable from village amenities file
Other_water_Status	Others (Status A(1)/NA(2))	Derived variable from village amenities file
Closed_Drainage_Status	Closed Drainage (Status A(1)/NA(2))	Derived variable from village amenities file
Open_Drainage_Status	Open Drainage (Status A(1)/NA(2))	Derived variable from village amenities file
No_Drainage_Status	No Drainage (Status A(1)/NA(2))	Derived variable from village amenities file
Open_Pucca_Drainage_Covered_with	Open Pucca Drainage Covered with Tiles Slabs (Status A(1)/NA(2))	Derived variable from village amenities file
Open_Pucca_Drainage_Uncovered__S	Open Pucca Drainage Uncovered (Status A(1)/NA(2))	Derived variable from village amenities file
Open_Kuccha_Drainage__Status_A_1	Open Kuccha Drainage (Status A(1)/NA(2))	Derived variable from village amenities file
Whether_Drain_water_is_discharge	Whether Drain water is discharged directly into water bodies or to sewar plant (	Derived variable from village amenities file
TSC_coverage	Is the Area Covered under Total Sanitation Campaign (TSC)? (Status A(1)/NA(2))	Derived variable from village amenities file
Community_Toilet_Complex__includ	Community Toilet Complex (including Bath) for General Public (Status A(1)/NA(2))	Derived variable from village amenities file
Community_Toilet_Complex__exclud	Community Toilet Complex (excluding Bath) for General Public (Status A(1)/NA(2))	Derived variable from village amenities file
Rural_Production_Centres_or_Sani	Rural Production Centres or Sanitary hardware outlet availability near the village (Status A(1)/NA(2))	Derived variable from village amenities file

Rural_Production_mart_or_Sanitar	Rural Production mart or Sanitary hardware outlet availability near the village (Status A(1)/NA(2))	Derived variable from village amenities file
Community_waste_disposal_system	Community waste disposal system after house to house collection (Status A(1)/NA(2))	Derived variable from village amenities file
Community_Bio_gas_or_recycle_of	Community Bio-gas or recycle of waste for production use (Status A(1)/NA(2))	Derived variable from village amenities file
No_System__Garbage_on_road_stree	No System (Garbage on road/street) (Status A(1)/NA(2))	Derived variable from village amenities file
Post_Office_Status	Post Office (Status A(1)/NA(2))	Derived variable from village amenities file
Sub_Post_Office_Status	Sub Post Office (Status A(1)/NA(2))	Derived variable from village amenities file
Post_And_Telegraph_Office__Statu	Post And Telegraph Office (Status A(1)/NA(2))	Derived variable from village amenities file
Village_Pin_Code_Status	Village Pin Code (Status A(1)/NA(2))	Derived variable from village amenities file
Telephone_Landlines_Status	Telephone (landlines) (Status A(1)/NA(2))	Derived variable from village amenities file
PCO_Mobile_Status	Public Call Office /Mobile (PCO) (Status A(1)/NA(2))	Derived variable from village amenities file
Mobile_Phone_Coverage__Status_A_	Mobile Phone Coverage (Status A(1)/NA(2))	Derived variable from village amenities file
Internet_Cafes___Common_Service	Internet Cafes / Common Service Centre (CSC) (Status A(1)/NA(2))	Derived variable from village amenities file
Private_Courier_Facility__Status	Private Courier Facility (Status A(1)/NA(2))	Derived variable from village amenities file
Public_Bus_Service_Status	Public Bus Service (Status A(1)/NA(2))	Derived variable from village amenities file
Private_Bus_Service_Status	Private Bus Service (Status A(1)/NA(2))	Derived variable from village amenities file
Railway_Station_Status	Railway Station (Status A(1)/NA(2))	Derived variable from village amenities file
Auto_Status	Auto/Modified Autos (Status A(1)/NA(2))	Derived variable from village amenities file
Taxi_Status	Taxi (Status A(1)/NA(2))	Derived variable from village amenities file
Vans_Status	Vans (Status A(1)/NA(2))	Derived variable from village amenities file
Tractors_Status	Tractors (Status A(1)/NA(2))	Derived variable from village amenities file
Cycle_pulled_Rickshaws____manual	Cycle-pulled Rickshaws (manual driven) (Status A(1)/NA(2))	Derived variable from village amenities file
Cycle_pulled_Rickshaws__machine	Cycle-pulled Rickshaws (machine driven) (Status A(1)/NA(2))	Derived variable from village amenities file
Carts_Drivens_by_Animals__Status	Carts Drivens by Animals (Status A(1)/NA(2))	Derived variable from village amenities file
Sea_River_Ferry_Service_Status	Sea/River/Ferry Service (Status A(1)/NA(2))	Derived variable from village amenities file
National_Highway_Status	National Highway (Status A(1)/NA(2))	Derived variable from village amenities file
State_Highway_Status	State Highway (Status A(1)/NA(2))	Derived variable from village amenities file

MajorDistrict_Highway_Status	Major District Road (Status A(1)/NA(2))	Derived variable from village amenities file
OtherDistrict_Highway_Status	Other District Road (Status A(1)/NA(2))	Derived variable from village amenities file
Pucca_Road_Status	Black Topped (pucca) Road (Status A(1)/NA(2))	Derived variable from village amenities file
Gravel_Road_Status	Gravel (kuchha) Roads (Status A(1)/NA(2))	Derived variable from village amenities file
Water_Bounded_Macadam__Status_A_	Water Bounded Macadam (Status A(1)/NA(2))	Derived variable from village amenities file
AllWeather_Road_Status	All Weather Road (Status A(1)/NA(2))	Derived variable from village amenities file
Navigable_Waterways__River_Canal	Navigable Waterways (River/Canal) (Status A(1)/NA(2))	Derived variable from village amenities file
Footpath_Status	Foothpath (Status A(1)/NA(2))	Derived variable from village amenities file
ATM_Status	ATM (Status A(1)/NA(2))	Derived variable from village amenities file
Commercial_Bank_Status	Commercial Bank (Status A(1)/NA(2))	Derived variable from village amenities file
Cooperative_Bank_Status	Cooperative Bank (Status A(1)/NA(2))	Derived variable from village amenities file
Agricultural_Credit_Societies__S	Agricultural Credit Societies (Status A(1)/NA(2))	Derived variable from village amenities file
Self_Help_Group_Status	Self - Help Group (SHG) (Status A(1)/NA(2))	Derived variable from village amenities file
Public_Distribution_System__PDS_	Public Distribution System (PDS) Shop (Status A(1)/NA(2))	Derived variable from village amenities file
Regular_Market_Status	Mandis/Regular Market (Status A(1)/NA(2))	Derived variable from village amenities file
Weekly_Haat_Status	Weekly Haat (Status A(1)/NA(2))	Derived variable from village amenities file
Agricultural_Marketing_Society__	Agricultural Marketing Society (Status A(1)/NA(2))	Derived variable from village amenities file
Nutritional_Centres_ICDS__Status	Nutritional Centres-ICDS (Status A(1)/NA(2))	Derived variable from village amenities file
Nutritional_Centres_Anganwadi_Ce	Nutritional Centres-Anganwadi Centre (Status A(1)/NA(2))	Derived variable from village amenities file
Nutritional_Centres_Others__Stat	Nutritional Centres-Others (Status A(1)/NA(2))	Derived variable from village amenities file
ASHA_Status	ASHA (Status A(1)/NA(2))	Derived variable from village amenities file
Community_Centre_with_without_TV	Community Centre with/without TV (Status A(1)/NA(2))	Derived variable from village amenities file
Sports_Field_Status	Sports Field (Status A(1)/NA(2))	Derived variable from village amenities file
Sports_Club_Recreation_Centre__S	Sports Club/Recreation Centre (Status A(1)/NA(2))	Derived variable from village amenities file
Cinema_Video_Hall	Cinema/Video Hall (Status A(1)/NA(2))	Derived variable from village amenities file
Public_library_status	Public Library (Status A(1)/NA(2))	Derived variable from village amenities file
Public_reading_room	Public Reading Room (Status A(1)/NA(2))	Derived variable from village amenities file
Daily_Newspaper_Supply__Status_A	Daily Newspaper Supply (Status A(1)/NA(2))	Derived variable from village amenities file

Assembly_Polling_Station__Status	Assembly Polling Station (Status A(1)/NA(2))	Derived variable from village amenities file
Birth_and_Death_Registration_Off	Birth and Death Registration Office (Status A(1)/NA(2))	Derived variable from village amenities file
Power_Supply_For_Domestic_Use	Power Supply For Domestic Use (Status A(1)/NA(2))	Derived variable from village amenities file
Power_Supply_For_Agriculture_Use	Power Supply For Agriculture Use (Status A(1)/NA(2))	Derived variable from village amenities file
Power_Supply_For_Commercial_Use	Power Supply For Commercial Use (Status A(1)/NA(2))	Derived variable from village amenities file
Power_Supply_For_All_Users	Power Supply For All Users (Status A(1)/NA(2))	Derived variable from village amenities file
Forest_Area__in_Hectares_	Forest Area (in Hectares)	Raw variable from village amenities file
Area_under_Non_Agricultural_Uses	Area under Non-Agricultural Uses (in Hectares)	Raw variable from village amenities file
Barren___Un_cultivable_Land_Area	Barren & Un-cultivable Land Area (in Hectares)	Raw variable from village amenities file
Permanent_Pastures_and_Other_Gra	Permanent Pastures and Other Grazing Land Area (in Hectares)	Raw variable from village amenities file
Land_Under_Miscellaneous_Tree_Cr	Land Under Miscellaneous Tree Crops etc. Area (in Hectares)	Raw variable from village amenities file
Culturable_Waste_Land_Area__in_H	Culturable Waste Land Area (in Hectares)	Raw variable from village amenities file
Fallows_Land_other_than_Current	Fallows Land other than Current Fallows Area (in Hectares)	Raw variable from village amenities file
Current_Fallows_Area__in_Hectare	Current Fallows Area (in Hectares)	Raw variable from village amenities file
Net_Area_Sown__in_Hectares_	Net Area Sown (in Hectares)	Raw variable from village amenities file
Total_Unirrigated_Land_Area__in	Total Unirrigated Land Area (in Hectares)	Raw variable from village amenities file
Area_Irrigated_by_Source__in_Hec	Area Irrigated by Source (in Hectares)	Raw variable from village amenities file
Canals_Area__in_Hectares_	Canals Area (in Hectares)	Raw variable from village amenities file
Wells_Tube_Wells_Area__in_Hectar	Wells/Tube Wells Area (in Hectares)	Raw variable from village amenities file
Tanks_Lakes_Area_Hectares	Tanks/Lakes Area (in Hectares)	Raw variable from village amenities file
Waterfall_Area__in_Hectares_	Waterfall Area (in Hectares)	Raw variable from village amenities file
Other_Area	Other Source (specify) Area (in Hectares)	Raw variable from village amenities file
Nearest_Town_Name	Nearest Town Name	Raw variable from village amenities file
Nearest_Town_Distance_from_Villa	Nearest Town Distance from Village (in Km.)	Raw variable from village amenities file

Dataset S1. (separate file)

Predicted estimates of child anthropometric failures for 597,121 villages

Dataset S2. (separate file)

Analytic codes used for prediction modeling
